# Adaptation and Constraint in the Atypical Chemokine Receptor Family in Mammals

**DOI:** 10.1155/2018/9065181

**Published:** 2018-09-24

**Authors:** Li Pan, Jianliang Lv, Zhongwang Zhang, Yongguang Zhang

**Affiliations:** ^1^State Key Laboratory of Veterinary Etiological Biology, National Foot-and-Mouth Disease Reference Laboratory, Lanzhou Veterinary Research Institute, Chinese Academy of Agricultural Sciences, No.1 Xujiaping, Yangchangbu, Chengguan District, Lanzhou 730046, Gansu, China; ^2^Jiangsu Co-Innovation Center for Prevention and Control of Important Animal Infectious Diseases and Zoonoses, Yangzhou, Jiangsu, China

## Abstract

Atypical chemokine receptors (ACKRs) are a subclass of G protein-coupled receptors characterized by promiscuity of ligand binding and an obvious inability to signal after ligand binding. Although some discoveries regarding this family in* Homo sapiens* and other species have been reported in some studies, the evolution and function of multiple ACKR in mammals have not yet been clearly understood. We performed an evolutionary analysis of* ACKR* genes (*ACKR1*,* ACKR2*,* ACKR3*, and* ACKR4*) in mammals. Ninety-two full-length* ACKR* genes from 27 mammal species were retrieved from the Genbank and Ensemble databases. Phylogenetic analysis showed that there were four well-conserved subfamilies in mammals. Synteny analysis revealed that* ACKR* genes formed conserved linkage groups with their adjacent genes across mammalian species, facilitating the identification of* ACKRs* in as yet unannotated genome datasets. Analysis of the site-specific profiles established by posterior probability revealed the positive-selection sites to be distributed mainly in the ligand binding region of ACKR1. This study highlights the molecular evolution of the* ACKR* gene family in mammals and identifies the critical amino acid residues likely to be relevant to ligand binding. Further experimental verification of these findings may provide valuable information regarding the ACKR's biochemical and physiological functions.

## 1. Introduction

The chemokine system plays an important role in mammalian immunity, which can guide immune effector cells to sites of infection or inflammation and coordinate interactions between immune cells. The chemokine family is classified into four main subfamilies (XC, CC, CXC, CX3C) based on the positioning of their initial cysteine residues (here C indicates cysteine and X/X3 indicates one or there non-cysteine amino acids) [1]. All of chemokines exert their functions by interacting with chemokine receptors that are selectively expressed on the surfaces of their target immune cells [2]. Chemokine receptors belong to the largest rhodopsin family of G protein-coupled receptors (GPCRs) and they consist of seven transmembrane domains and multiple extracellular and intracellular loops. These are involved in ligand binding and signaling [3]. Chemokine receptors are mainly divided into four subfamilies depending on the type of chemokine they bind, including CC chemokine receptors (CCRs), CXC chemokine receptors (CXCRs), XC chemokine receptors (XCRs), and CX3C chemokine receptors (CX3CRs) [4]. Apart from signaling receptors, chemokine receptors also include some atypical receptors. These are similar in structure to the conventional receptors, but lack an intracellular motif required for signaling [5]. The conventional chemokine receptors represent a larger subgroup (eighteen in humans) of G protein coupled leukocyte chemotactic receptors, and the atypical chemokine receptors represent a smaller subgroup (four in humans) of chemotactic receptors that do not transduce signals through G proteins and lack chemotactic activity [6]. Atypical chemokine receptors mainly include four types of receptors and named atypical chemokine receptor 1 (ACKR1), ACKR2, ACKR3, and ACKR4 on the basis of the new standardized nomenclature system [7]. Members of ACKR have been previously referred to by scientists in this filed as ‘chemokine-binding proteins', ‘scavengers', ‘interceptors', and ‘decoys', which has caused confusion and has delayed recognition of these molecules as a functionally related groups. For instance, ACKR1 is previously known as Duffy antigen, Fy antigen, and CD234. ACKR2 is previously known as D6, CCBP2, CCR9, and CMKBR9. ACKR3 is previously known as CXCR7 and RDC1. ACKR4 is previously known as CCRL1 and CCX-CKR.

Atypical chemokine receptors are characterized by promiscuity of ligand binding and an apparent inability to signal after ligand binding. This inability to signal is mainly because of alterations in the canonical DRY motif located in the second intracellular loop of the typical chemokine receptors [8]. This motif is responsible for G protein coupling to the receptors and its absence is an essential unifying theme of the atypical receptors [9, 10]. These nonsignaling receptors efficiently internalize their cognate chemokines and act as chemokine scavengers. This is achieved by continuous internalization and constant surface expression of the atopic receptors in a *β*-arrestin signaling dependent manner [11–13].

ACKR1 is mainly expressed in red blood cells and endothelial cells and has very little sequence similarity to other atypical receptors or chemokine receptors [14, 15].* ACKR* genes exist in a specific chromosomal location, unlike those of the other receptors [15]. The ligand binding profile of ACKR1 includes several inflammatory chemokines, including CXCL1, CXCL5-9, CXCL11, and CXCL13, suggesting that ACKR1 plays an important role in maintaining chemokine receptors in the blood [16, 17]. ACKR2 is expressed in many different tissues including those of the skin, gut, and lung [18]. Within these classical barrier tissues, expression is largely confined to lymphatic endothelial cells, with no expression detected in blood endothelial cells [18]. The ligand binding profile includes CCL2-5, CCL7-8, CCL11, CCL13-14, CCL17, and CCL22 [19–24]. ACKR2, which serves as a constitutively internalizing and recycling receptor, is also capable of internalizing and degrading the chemokines of its binding profile [25]. ACKR3 is the best characterized receptor in the ACKR family and it is mainly expressed in hematopoietic cells, neurons, mesenchymal cells, endothelial cells, and cancer cells. The ligands of ACKR3 contain CXCL11 and CXCL12. Binding of ACKR3 to CXCL11, which is an IFN-*γ*-induced chemokine, is involved in the regulation of lymphocyte migration [26]. ACKR3 also forms a heterodimer with CXCR4 and acts as a scavenger for the ligand CXCL12 and plays key roles in organ development and tumor development and progression [27]. ACKR4 is an atypical receptor for homeostatic CC and CXC chemokines including CCL19, CCL21, CCL25, and CXCL13 [28]. Like ACKR3, ACKR4 is capable of internalizing its ligands and targeting them for intracellular degradation, and it does so in much the same way. ACKR4 is also able to antagonize CXCR3-induced chemokines through heterodimer formation with the CXCR3 [29].

Two other ACKRs, CCRL2 (ACKR5) and PITPNM3 (ACKR6), have been proposed, but functional confirmation is pending, so they have been provisionally assigned ACKR designations [30].

Compared with numerous studies on typical chemokine receptors, especially in fish and mammals, little is known about ACKRs in mammals [30]. The increasing wealth of sequence data available from sequenced genome databases has allowed researchers to perform evolutionary analyses of ACKRs in mammals. In the present study, we performed an evolutionary analysis of 92 full-length* ACKR* (*ACKR1*,* ACKR2*,* ACKR3*, and* ACKR4*) genes from 27 mammal species retrieved from the Genbank and Ensemble databases.

## 2. Materials and Methods

### 2.1. Acquisition of ACKR Family Sequences and Synteny Analysis

For some well-annotated genomes, the amino acid sequences of ACKRs (*ACKR1*,* ACKR2*,* ACKR3*, and* ACKR4*) were retrieved directly from the Genbank and Ensemble databases. PSI-BLAST was performed to search these databases using* Homo sapiens* ACKRs (NP_001136269, NP_001008540, NP_001707, and NP_006555) as query sequences. The predicted coding sequences of the best hits were collected when the hits presented more than 70% in length and 50% in identity were aligned with the query sequence (with E values < e^−10^). These settings distinguished the potential ACKR members from different species but avoid involving other chemokine receptors effectively. After removal of redundant and incomplete sequences, the initial data set (S1 Table) for ACKR contained 92 protein sequences from 27 mammals. Because the functional confirmation of the two provisionally assigned ACKRs (ACKR5 and ACKR6) is pending, they were excluded from analysis in the present study.

Synteny analysis was conducted using the GENOMICUS v80.01 browser, which allows integration of the data available on the Ensemble database to provide a better visualization of conserved synteny blocks and to facilitate reconstruction of the organization of ancient genomes [31, 32]. Genes not annotated on the GENOMICUS browser were searched within the respective species by BLASTP and TBLASTN over the Genbank and Ensemble databases.

### 2.2. Sequence Alignment and Phylogenetic Analysis

A codon-based coding sequence alignment was constructed using MUSCLE with default parameters and manually adjusted using MEGA 6 [33] and viewed and edited in Jalview 2.0 [34]. The alignment was subsequently processed using Gblocks v0.91b [35] for phylogenetic reconstruction with default parameters. To access the selective pressures acting on the four mammals ACKR subfamilies, seven different alignments were produced: one for each paralog and a seventh with all sequences excluding outgroups. The substitution model that best fit the dataset was selected using Akaike Information Criterion (AIC) implemented in ProTest 3.2 [36], starting with 14 substitution matrices and using the fixed BIONJ tree for likelihood calculations. The phylogeny was estimated using the Maximum Likelihood (ML) methods. The ML phylogenetic tree was constructed in PhyML 3.0 [37], with 1000 bootstrap replicates and the NNI branch search algorithm. Finally, the phylogenetic trees were displayed using TreeView [38]. Besides, the neighbor joining (NJ), minimal evolution (ME) and maximum parsimony (MP) methods were used individually to reconstruct another three phylogenetic trees with MEGA v3.1 from the Gblocks alignment.

### 2.3. Codon-Based Analyses of Positive Selection

The selective pressures acting on coding region were evaluated across the phylogeny using a phylogenetic-based ML analysis. Accurate nucleotide sequences and related amino acid sequence alignments were retrieved with PAL2NAL [39], a program that constructs multiple codon alignments form matching protein sequences. The* codeml* program in PAML4.5 [40] was used to estimate the rates of synonymous (*dS*) and nonsynonymous substitution (*dN*) and the* dN/dS* ratio (omega, *ω*). *ω*>1 indicates positive selection, *ω*<1 indicates negative selection, and *ω*=1 is neutrality. Accurate nucleotide sequence alignments were constructed from matching related protein sequence with MUSCLE (MEGA 6). Then the resulting codon alignments and ML tree were used in the* codeml* program. The site-specific models were tested: Models M0 (one ratio), M1a (nearly neutral), M2a (positive selection), M3 (discrete), M7 (beta), and M8 (beta+*ω*) were all used in this analysis [41, 42]. Model M0 assumes one ratio for all sites. M1a presupposes a proportion p0 of conserved sites with *ω*<1 and p1=1-p0 of neutral sites with *ω*=1. M2a adds an additional class of sites with the frequency p2=1-p0-p1, and *ω*2 is estimated form the data. In the M3, the probabilities (p0, p1, and p2) of each site being submitted to purifying, neutral, and positive selection, respectively, and their corresponding *ω* ratios (*ω*0, *ω*1, and *ω*2) are inferred from the data. M7 and M8 assume a *β*-distribution for *ω* between 0 and 1, and M8 adds one extra class with the same ratio *ω*1. Subsequent likelihood rate comparisons of M0 and M3, M1a with M2a, and M7 with M8 were performed to determine which model fit the data best. The LRT was used to test positive selection of the two pairs of site model [43, 44]. Finally, the BEB approach was used to calculate the posterior probability that each site would belong to the site class of positive selection under each model [45].

## 3. Results

### 3.1. Identification and Distribution of* ACKR* Genes across Mammals

The final data sets contained 92* ACKR* gene sequences from 27 representative species of mammals, including two primates, two rodents, one monotreme, one hyracoidean, one edentate, one pilosa, two perissodactyls, three artiodactyls, two carnivorans, one cetacean, one proboscid, two erinaceidae, one insectivoran, two lagomorphs, one dasyuromorph, one scandentia, one diprotodont, and two chiropterans. The results of genomic database searches showed that the majority of mammals investigated in this study (from orders Primate, Rodentia, Edentata, Perissodactyla, Carnivora, Proboscidea, Erinaceidae, Insectivora, Lagomorpha, Dasyuromorphia, Chiroptera, Artiodactylas) possess 4 members of* ACKR* family. No* ACKR* genes were identified in the representative of Pilosa,* Choloepus hoffmanni*. The remaining mammals only possessed 2* ACKRs* and all of these species lacked the* ACKR1 *gene (S1 Table).

### 3.2. Synteny Analysis of* ACKR* Genes in Mammal Genomes

**Figure 1:**
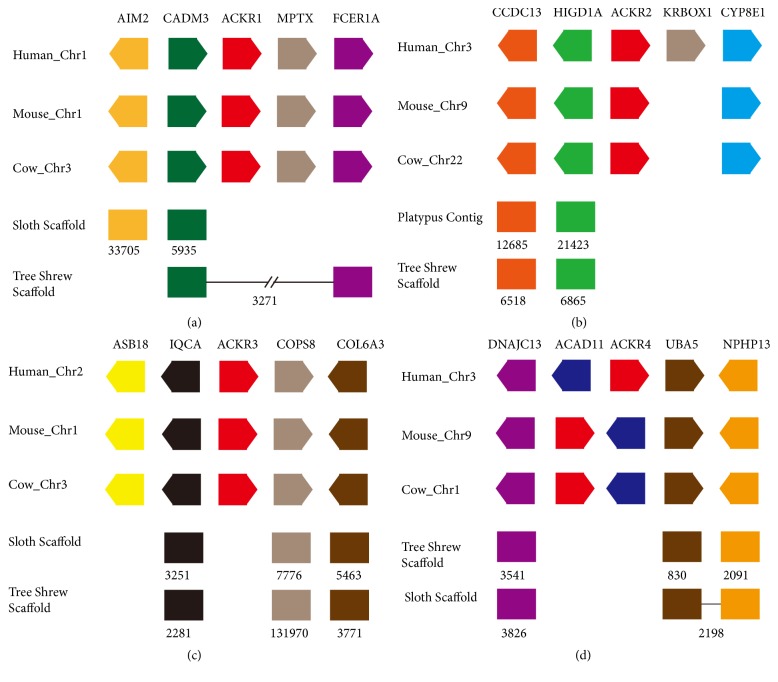
*Gene synteny analysis of ACKR1-4 in representative species of mammals.* The gene abbreviations were taken from the Ensemble database. The directions of arrows indicate the transcriptional orientation of genes.

As shown in the additional file 1, several* ACKR* genes could not be identified in some mammal genomes using the sequence collection method. Synteny analysis was performed to determine why some* ACKR* genes were missed. We observed that the* ACKR1* gene formed a conserved linkage group with* AIM2*,* CADM3*, and* FCER1A* genes in the most mammal genomes (Figure 1(a)). In the genome databases of* C. hoffmanni*, only two genes of the conserved linkage group, the* AIM2* and* CADM3*, were found. These were located in Scaffold_33705 and Scaffold_ 5395, respectively.* CADM3* and* FCER1A*, which were in the conserved group, were identified in the genomes of* Tupaia belangeri *but* AIM2* and* ACKR1* were not. In the genome of* Ornithorhynchus anatinus*, no members of the conserved linkage group were found.* ACKR2* formed a conserved linkage group with* CCDC13*,* HIGD1A* and* CYP8B1* in mammalian genomes (Figure 1(b)). However, no* ACKR2* genes were found in the genomes of* C. hoffmanni* or* O. anatinus*. Only* CCDC13* and* HIGD1A* of the conserved gene group were identified. The conserved* ACKR3*-specific gene group consisted of* ASB18, IQCA, ACKR3, COPS8*, and* COL6A3* in mammal genomes (Figure 1(c)). Nevertheless, neither* ASB18* nor* ACKR3 *were found in the genome of* C. hoffmanni *or* T. belangeri*. In mammalian genomes, the* ACKR4*-specific conserved linkage group was composed of* ACPP, DNAJC13, ACAD11, ACKR4, UBA5* and* NPHP5* (Figure 1(d)). Among these genes,* AKCR4, ACAD11, UBA5* and* NAHP* shared the same transcript: “*ACAD11-NPHP5*”. The* ACAD11* and* ACKR4* of this conserved gene group were absent from the genomes of* C. hoffmanni *and* T. belangeri*.

### 3.3. Phylogenetic Analysis of* ACKR* Genes in Mammals

**Figure 2:**
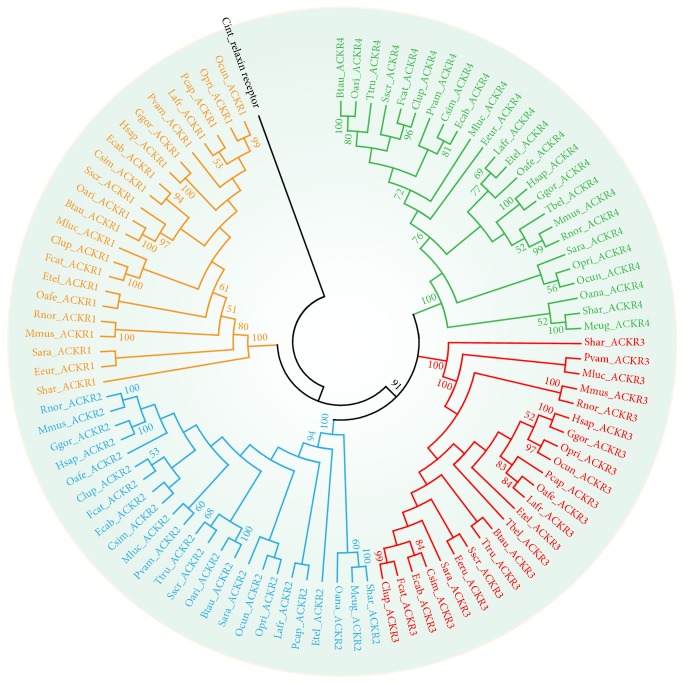
*A maximum likelihood tree of mammal ACKRs.* The tree was constructed using maximum likelihood method. The number indicates bootstrap values. See additional file 1 for details of gene accession numbers and species abbreviations.

After the exclusion of partial and unfinished sequences, 92 sequences were retrieved from 27 mammal species. To determine the phylogenetic relationship of mammal* ACKR* genes, a rooted ML phylogenetic tree was constructed based on amino acids alignment under the best-fit model JTT+I+G+F. Here, the best-fit model (JTT+I+G+F) for amino acid substitution was selected by ProTest3.2 with discrete gamma distribution in four categories. All parameters (gamma shape = 1.687; proportion of invariants = 0.042) were estimated from the dataset. Tree topology was assessed using MEGA 6 with neighbor joining (NJ), minimum evolution (ME) and maximum parsimony (MP) methods, and it was found to be substantially similar to the ML tree (data not shown). Using the relaxin receptor from* Ciona intestinalis* as the outgroup of mammal* ACKR* genes [30], the ML trees showed the* ACKRs* of mammals to be grouped into four lineages:* ACKR1* subfamily,* ACKR2* subfamily,* ACKR3* subfamily and* ACKR4* subfamily (Figure 2). Our data suggested that two major duplications had occurred in mammal lineages. The first duplication led to the emergence of two lineages that evolved into* ACKR1 *and the ancestor of* ACKR2*,* ACKR3* and* ACKR4*. The second duplication led to divergence of ACKR2, ACKR3 and ACKR4.

### 3.4. Adaptive Evolution of* ACKR* Genes in Mammals

To detect signatures of adaptive evolution over the* ACKR1, ACKR2, ACKR3*, and* ACKR4 *codon sequences, four smaller phylogenetic trees were built for each group and the topology used for each site-specific model was implanted using the* codeml* program of PAML v4.0 package. Parameter estimates and log-likelihood values under model of variable *ω* ratios among sites were shown in Table 1. In all cases, the LRT did not differ significantly between M1a and M2a, but the LRTs did show significant differences between M0 and M3 and between M7 and M8 for all receptors except the* ACKR4* lineage, indicating that M3 and M8 fit the data better. However, no selected sites were detectable in M3. In model M8, one site (154 G) from* ACKR1* lineage was found to be a positively selected site, showing a* P*-value over 99%.

## 4. Discussion

**Figure 3:**
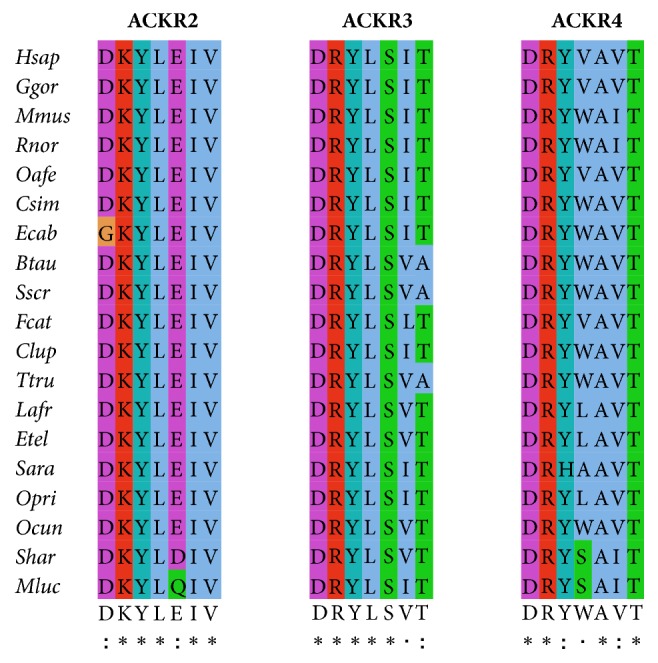
*Sequence alignment of the DRY motif in the ACKR2, ACKR3, and ACKR4 proteins of mammals.* Multiple alignments were performed using the full length protein sequences with the Jalview software. Identical amino acids are indicated by asterisks whereas those with high or low similarity are indicated by “:” and “.” respectively.

Chemokines are important regulators of leukocyte migration and play key roles in diverse physiological and pathological immune and inflammatory contexts [28]. In addition to the typical signaling chemokine receptors, a recently discovered subclass of atypical chemokine receptors are characterized by promiscuity of ligand binding and an obvious inability to signal after ligand binding [46]. The inability to signal is largely a consequence of alterations in the canonical DRY motif in the second intracellular loop of the typical chemokine receptors [47]. The motif is responsible for G-protein coupling to the receptors and its absence is the key unifying theme of these atypical receptors [8]. The DRY motif of the ACKRs was identified using multiple sequence alignment. No DRY motif was found in the ACKR1 subgroup. The DKYLEIV motif, DRYLSVT motif, and DRYWAVT motif were identified in the mammal ACKR2, ACKR3, and ACKR4 subgroups, respectively. As in the DRY motif of typical chemokine receptors, the last three amino acids were essential to maintaining the function of signal transduction [8, 48] (Figure 3).

The number of the* ACKR* genome loci varied across several mammalian genomes. Synteny analysis was performed to determine the reason for the absence of some* ACKR* genes.* ACKR* genes formed conserved linkage groups with their adjacent genes across mammalian genomes. The genome sequence datasets of* O. anatinus*,* P. capensis*, and* C. hoffmanni* available in Genbank and Ensemble databases were limited and presented in scaffold form. These sequence data did not meet the requirements for assembly into chromosomes. Partial segments of the conserved* ACKR*-specific blocks of genes were here identified. In this way, the absence of* ACKRs* from some of the mammalian genomes investigated here may be attributed to the incomplete information available in genome databases rather than to gene loss during evolution.

**Figure 4:**
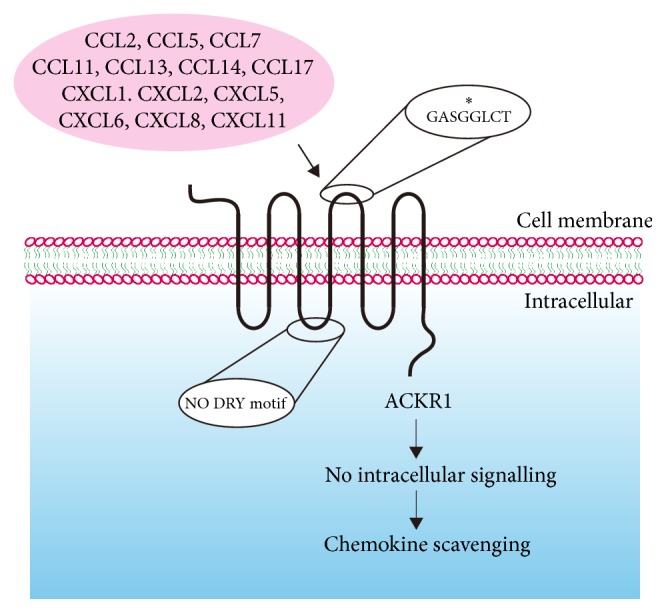
*The ACKR1 receptors do not signal in response to ligand binding but rather internalize the ligand and target it for intracellular degradation.* The key to the lack of signaling is the absence of DRY motif in the second intracellular loop of ACKR1. The positively selective site was located in the second extracellular loop, which is responsible for ligand recognition and binding.

The positively selective sites 154 G of ACKR1 is located within the extracellular domain between the fourth trans-membrane and the fifth trans-membrane (Figure 4). This region is responsible for the direct interaction between ACKR1 and the ligands [15]. ACKR1 is mainly expressed in red blood cell. ACKR1 serves as the chemokine buffer for the blood, and it can bind to many different chemokines. Increasing amounts of evidence have shown that ACKR1 possesses a larger ligand binding profile than the other ACKRs [49, 50]. The positively selective site within the binding region of ACKR1 may provide direct evidence for extended ligand binding profile.

## Figures and Tables

**Table 1:** Likelihood ratio test statistics for models of variable selective pressure among sites.

Gene	Model	Parameters	Models compared	LRT(2Δl)	*P*-value	*df*	Positively selected sites (BEB)
ACKR1	M0	*ω*_0_=0.43574	M0 vs M3	534.02	**0.0000**	4	
	M3	*ω*_0_=0.03476 *ω*_1_= 0.35759 *ω*_2_= 1.15972					
		p_0_= 0.23127 p_1_= 0.47276 p_2_= 0.29597					
	M1a	*ω*_0_=0.16327 *ω*_1_=1.00000	M1a vs M2a	0	1.0000	2	
		p_0_=0.55005 p_1_=0.44995					
	M2a	*ω*_0_=0.16327 *ω*_1_= 1.00000 *ω*_2_=1.00000					
		p_0_=0.55005 p_1_=0.28372 p_2_=0.16623					
	M7	p =0.53074 q=0.60339	M7 vs M8	21.26	**0.0000**	2	154 G^*∗∗*^
	M8	p_0_=0.85617 p=0.68793 q=1.15451					
		p_1_=0.14383 *ω*=1.45019					
ACKR2	M0	*ω*_0_=0.20556	M0 vs M3	652.48	**0.0000**	4	
	M3	*ω*_0_=0.01987 *ω*_1_=0.21352 *ω*_2_= 0.77475					
		p_0_= 0.34607 p_1_=0.48100 p_2_= 0.17293					
	M1a	*ω*_0_=0.12737 *ω*_1_=1.00000	M1a vs M2a	0	1.0000	2	
		p_0_=0.79265 p_1_=0.20735					
	M2a	*ω*_0_=0.12737 *ω*_1_= 1.00000 *ω*_2_=1.00000					
		p_0_=0.79265 p_1_=0.18741 p_2_=0.01994					
	M7	p =0.52179 q=1.61923	M7 vs M8	8.88	**0.0117**	2	
	M8	p_0_=0.97423 p=0.59529 q=2.13409					
		p_1_=0.02577 *ω*=1.39256					
ACKR3	M0	*ω*_0_=0.04691	M0 vs M3	569.64	**0.0000**	4	
	M3	*ω*_0_=0.00786 *ω*_1_=0.12501 *ω*_2_= 0.49733					
		p_0_= 0.71583 p_1_=0.24584 p_2_=0.03833					
	M1a	*ω*_0_=0.03806 *ω*_1_=1.00000	M1a vs M2a	0	1.0000	2	
		p_0_=0.95014 p_1_=0.04986					
	M2a	*ω*_0_=0.03806 *ω*_1_= 1.00000 *ω*_2_=28.52785					
		p_0_=0.95014 p_1_=0.04986 p_2_=0.00000					
	M7	p =0.24148 q=3.44384	M7 vs M8	7.78	**0.0204**	2	
	M8	p_0_=0.98766 p=0.27944 q=5.15904					
		p_1_=0.01234 *ω*=1.00000					
ACKR4	M0	*ω*_0_=0.12972	M0 vs M3	416.55	**0.0000**	4	
	M3	*ω*_0_=0.03081 *ω*_1_=0.32186 *ω*_2_=1.16947					
		p_0_= 0.65355 p_1_=0.33146 p_2_=0.01499					
	M1a	*ω*_0_=0.08280 *ω*_1_=1.00000	M1a vs M2a	0	1.0000	2	
		p_0_=0.84629 p_1_=0.15371					
	M2a	*ω*_0_=0.08280 *ω*_1_= 1.00000 *ω*_2_=31.41467					
		p_0_=0.84629 p_1_=0.15371 p_2_=0.00000					
	M7	p =0.41473 q=2.39665	M7 vs M8	5.54	0.0626	2	139 V^*∗*^
	M8	p_0_=0.99543 p=0.43468 q=2.65383					
		p_1_=0.00457 *ω*=1.77610					

## Data Availability

The sequences of ACKR family analyzed in this study are deposited in the NCBI and Ensemble databases. The accession number is listed in Supplementary file 1.

## Supplementary Materials

Supplementary Materials Table S1. List of species used for the Multiple Sequences Alignment (MSA).
