# Supplementary material for: Adaptation and Constraint in the Atypical Chemokine Receptor Family in Mammals
Source: Biomed Res Int. 2018 Sep 24;2018:9065181. doi: 10.1155/2018/9065181 (PMC6174752; doi:10.1155/2018/9065181)
Supplement: Supplementary Materials — Table S1. List of species used for the Multiple Sequences Alignment (MSA). [file 9065181.f1.doc]

**Table S1. List of species used for the Multiple Sequences Alignment (MSA).**

| **Species** | **Abbreviation** | **Gene Symbol** | **Accession Number** | **Length^a^** | **Data Origin^b^** |
| --- | --- | --- | --- | --- | --- |
| **Primate** |  |  |  |  |  |
| *Homo sapiens* | Hsap | ACKR1 | NP _002027.2 | 336 | NCBI |
|  |  | ACKR2 | NP_001287.2 | 384 | NCBI |
|  |  | ACKR3 | NP_064707.1 | 362 | NCBI |
|  |  | ACKR4 | NP_057641.1 | 350 | NCBI |
| *Gorilla gorilla gorilla* | Ggor | ACKR1 | XP_004027105 | 336 | NCBI |
|  |  | ACKR2 | XP_004033980.1 | 384 | NCBI |
|  |  | ACKR3 | ENSGGOT00000004291 | 362 | Ensemble |
|  |  | ACKR4 | XP_004036368.1 | 350 | NCBI |
| **Rodent** |  |  |  |  |  |
| *Mus musculus* | Mmus | ACKR1 | NP_034175.2 | 334 | NCBI |
|  |  | ACKR2 | NP_067622.2 | 378 | NCBI |
|  |  | ACKR3 | NP_031748.2 | 362 | NCBI |
|  |  | ACKR4 | NP_663746.2 | 350 | NCBI |
| *Rattus norvegicus* | Rnor | ACKR1 | XP_002728079 | 331 | NCBI |
|  |  | ACKR2 | NP_511176.2 | 382 | NCBI |
|  |  | ACKR3 | NP_445804.1 | 362 | NCBI |
|  |  | ACKR4 | XP_006226609.1 | 350 | NCBI |
| **Monotremata** |  |  |  |  |  |
| *Ornithorhynchus anatinus* | Oana | CXCR1 | / | / | NCBI Ensemble |
|  |  | ACKR2 | / | / | NCBI Ensemble |
|  |  | ACKR3 | ENSOANT00000006332 | 363 | Ensemble |
|  |  | ACKR4 | XP_003430502.1 | 353 | NCBI |
| **Hyracoidea** |  |  |  |  |  |
| *Procavia capensis* | Pcap | ACKR1 | ENSPCAT00000013938 | 351 | Ensemble |
|  |  | ACKR2 | / | / | NCBI Ensemble |
|  |  | ACKR3 | ENSPCAT00000003090 | 361 | Ensemble |
|  |  | ACKR4 | / | / | NCBI Ensemble |
| **Edentata** |  |  |  |  |  |
| *Orycteropus afer afer* | Oafe | ACKR1 | XP_007945970.1 | 337 | NCBI |
|  |  | ACKR2 | XP_007947900.1 | 383 | NCBI |
|  |  | ACKR3 | XP_007939801 | 362 | NCBI |
|  |  | ACKR4 | XP_007935141.1 | 350 | NCBI |
| **Pilosa** |  |  |  |  |  |
| *Choloepus hoffmanni* | Chof | CXCR1 | / | / | NCBI Ensemble |
|  |  | ACKR2 | / | / | NCBI Ensemble |
|  |  | ACKR3 | / | / | NCBI Ensemble |
|  |  | ACKR4 | / | / | NCBI Ensemble |
| **Perissodactyla** |  |  |  |  |  |
| *Ceratotherium simum simum* | Csim | ACKR1 | XP_004442876.1 | 323 | NCBI |
|  |  | ACKR2 | XP_004419631.1 | 384 | NCBI |
|  |  | ACKR3 | XP_004427772 | 362 | NCBI |
|  |  | ACKR4 | XP_004419423.1 | 350 | NCBI |
| *Equus caballus* | Ecab | ACKR1 | XP_001490691.2 | 366 | NCBI |
|  |  | ACKR2 | XP_005600847.1 | 384 | NCBI |
|  |  | ACKR3 | NP_001244007 | 362 | NCBI |
|  |  | ACKR4 | XP_001917935.1 | 350 | NCBI |
| **Artiodactyla** |  |  |  |  |  |
| *Bos taurus* | Btau | ACKR1 | NP_001015634.1 | 360 | NCBI |
|  |  | ACKR2 | NP_001015581.1 | 384 | NCBI |
|  |  | ACKR3 | NP_001091851 | 362 | NCBI |
|  |  | ACKR4 | NP_776690.1 | 350 | NCBI |
| *Ovis aries* | Oari | ACKR1 | XP_004002694.1 | 330 | NCBI |
|  |  | ACKR2 | / | / | NCBI Ensemble |
|  |  | ACKR3 | XP_004001817 | 362 | NCBI |
|  |  | ACKR4 | XP_004003386.1 | 350 | NCBI |
| *Sus scrofa* | Sscr | ACKR1 | NP_001231024.1 | 341 | NCBI |
|  |  | ACKR2 | NP_001243702.1 | 385 | NCBI |
|  |  | ACKR3 | XP_003133807 | 362 | NCBI |
|  |  | ACKR4 | NP_001090899.1 | 350 | NCBI |
| *Tursiops truncatus* | Ttru | ACKR1 | / | / | NCBI Ensemble |
|  |  | ACKR2 | XP_019802503.1 | 391 | NCBI |
|  |  | ACKR3 | XP_004318379.1 | 362 | NCBI |
|  |  | ACKR4 | XP_019789741.1 | 350 | NCBI |
| **Carnivora** |  |  |  |  |  |
| *Felis catus* | Fcat1 | ACKR1 | XP_003999716.1 | 339 | NCBI |
|  | Fcat2 | ACKR2 | XP_006936557.1 | 386 | NCBI |
|  | Fcat3 | ACKR3 | XP_006935769 | 380 | NCBI |
|  | Fcat4 | ACKR4 | XP_003992166.1 | 350 | NCBI |
| *Canis lupus familiaris* | Clup1 | ACKR1 | XP_005641007.1 | 433 | NCBI |
|  | Clup2 | ACKR2 | XP_005634371.1 | 384 | NCBI |
|  | Clup3 | ACKR3 | NP_001003281 | 362 | NCBI |
|  | Clup4 | ACKR4 | XP_005634482.1 | 350 | NCBI |
| **Proboscidea** |  |  |  |  |  |
| *Loxodonta africana* | Lafr1 | ACKR1 | XP_010593165.1 | 304 | NCBI |
|  | Lafr2 | ACKR2 | XP_003409975.1 | 384 | NCBI |
|  | Lafr3 | ACKR3 | ENSLAFT00000003100 | 342 | Ensemble |
|  | Lafr4 | ACKR4 | XP_003420957.1 | 350 | NCBI |
| **Erinaceidae** |  |  |  |  |  |
| *Erinaceus europaeus* | Eeur1 | ACKR1 | XP_007530657.1 | 332 | NCBI |
|  | Eeur2 | ACKR2 | XP_007537604.1 | 378 | NCBI |
|  | Eeur3 | ACKR3 | XP_007537719.1 | 279 | NCBI |
|  | Eeur4 | ACKR4 | XP_007537647.1 | 350 | NCBI |
| *Echinops telfairi* | Etel1 | ACKR1 | XP_004714864.1 | 358 | NCBI |
|  | Etel2 | ACKR2 | XP_004708718.1 | 388 | NCBI |
|  | Etel3 | ACKR3 | XP_004701746 | 362 | NCBI |
|  | Etel4 | ACKR4 | XP_004716044.1 | 350 | NCBI |
| **Insectivora** |  |  |  |  |  |
| *Sorex araneus* | Sara1 | ACKR1 | XP_004613983.1 | 330 | NCBI |
|  | Sara2 | ACKR2 | XP_004614664.1 | 390 | NCBI |
|  | Sara3 | ACKR3 | XP_004610882 | 362 | NCBI |
|  | Sara4 | ACKR4 | XP_004603856.1 | 350 | NCBI |
| **Lagomorpha** |  |  |  |  |  |
| *Ochotona princeps* | Opri1 | ACKR1 | XP_004589320.1 | 343 | NCBI |
|  | Opri2 | ACKR2 | XP_004581773.1 | 383 | NCBI |
|  | Opri3 | ACKR3 | XP_004597461 | 362 | NCBI |
|  | Opri4 | ACKR4 | XP_004588375.1 | 349 | NCBI |
| *Oryctolagus cuniculus* | Ocun1 | ACKR1 | XP_002715354.1 | 338 | NCBI |
|  | Ocun2 | ACKR2 | XP_008258455.1 | 375 | NCBI |
|  | Ocun3 | ACKR3 | XP_002723286 | 363 | NCBI |
|  | Ocun4 | ACKR2 | XP_002716461.1 | 350 | NCBI |
| **Dasyuromorphia** |  |  |  |  |  |
| *Sarcophilus harrisii* | Shar1 | ACKR1 | XP_003767930.1 | 313 | NCBI |
|  | Shar2 | ACKR2 | ENSSHAT00000003965 | 371 | Ensemble |
|  | Shar3 | ACKR3 | ENSSHAT00000017351 | 362 | Ensemble |
|  | Shar4 | ACKR4 | XP_003772031.1 | 350 | NCBI |
| **Scandentia** |  |  |  |  |  |
| *Tupaia belangeri* | Tbel1 | CXCR1 | / | / | NCBI Ensemble |
|  | Tbel2 | ACKR2 | ENSTBET00000013899 | 303 | Ensemble |
|  | Tbel3 | ACKR3 | / | / | NCBI Ensemble |
|  | Tbel4 | ACKR4 | ENSTBET00000016063 | 313 | Ensemble |
| **Diprotodontia** |  |  |  |  |  |
| *Macropus eugenii* | Meug1 | CXCR1 | / | / | NCBI Ensemble |
|  | Meug2 | ACKR2 | / | / | NCBI Ensemble |
|  | Meug3 | ACKR3 | ENSMEUT00000008252 | 362 | Ensemble |
|  | Meug4 | ACKR4 | ENSMEUT00000003569 | 350 | Ensemble |
| **Chiroptera** |  |  |  |  |  |
| *Pteropus vampyrus* | Pvam1 | ACKR1 | XP_011371420.1 | 361 | NCBI |
|  | Pvam2 | ACKR2 | XP_011355788.1 | 429 | NCBI |
|  | Pvam3 | ACKR3 | ENSPVAT00000009504 | 362 | Ensemble |
|  | Pvam4 | ACKR4 | XP_011364366.1 | 350 | NCBI |
| *Myotis lucifugus* | Mluc1 | ACKR1 | XP_006096950.1 | 358 | NCBI |
|  | Mluc2 | ACKR2 | XP_006102358.1 | 418 | NCBI |
|  | Mluc3 | ACKR3 | ENSMLUP00000010501 | 362 | Ensemble |
|  | Mluc4 | ACKR4 | XP_006083764.1 | 350 | NCBI |

^a^Number of amino acid residues;

^b^NCBI: National Centre for Biotechnology Information; Ensemble: Ensemble Genome Browser.
